# Minimally Invasive Repair of Sinus Venosus Atrial Septal Defects and Anomalous Pulmonary Venous Connections via Vertical Right Axillary Thoracotomy

**DOI:** 10.3390/jcdd12100404

**Published:** 2025-10-11

**Authors:** Sameh M. Said, Ali H. Mashadi, Yasin Essa, Kristin Greathouse, Nicholas Brown, Mahmoud I. Salem, Joseph Giamelli

**Affiliations:** 1 Division of Pediatric and Adult Congenital Cardiac Surgery, Maria Fareri Children’s Hospital, Westchester Medical Center, New York Medical College, Valhalla, NY 10595, USA; ahmashadi01@gmail.com (A.H.M.);; 2 King Faisal Specialist Hospital and Research Center, Riyadh 11211, Saudi Arabia; 3 Department of Cardiothoracic Surgery, Faculty of Medicine, Alexandria University, Alexandria 21511, Egypt; 4 M Health Fairview Health System, Masonic Children’s Hospital, Minneapolis, MN 55455, USA; 5 Division of Pediatric Critical Care Medicine, Department of Pediatrics, Masonic Children’s Hospital, University of Minnesota, Minneapolis, MN 55455, USA; 6 Department of Cardiothoracic Surgery, Port Said University, Port Said 42614, Egypt; 7 Boston Children’s Health Physicians, Maria Fareri Children’s Hospital, Valhalla, NY 10595, USA; joseph_giamelli@bchphysicians.org

**Keywords:** minimally invasive, sinus venosus defects, anomalous pulmonary venous connections, vertical right axillary thoracotomy

## Abstract

(1) Background: There has been an increase in the utilization of the minimally invasive vertical right axillary thoracotomy approach for repairing congenital heart defects in children recently. We aim, in the current study, to evaluate the outcomes of this approach in repairing anomalous pulmonary venous connections with or without an associated sinus venosus defect. (2) Methods: A total of 23 consecutive patients underwent surgical repair of anomalous pulmonary venous connections between April 2018 and February 2024. Perioperative and clinical follow-up data were obtained. (3) Results: The median age and weight were 36 months (1–277 months) and 14.4 kg (3.6–79.4 kg), respectively. More than half were females (13; 56.5%). There was no conversion to sternotomy. Partial anomalous pulmonary venous connections were the most frequent primary diagnoses (14; 60.9%), followed by scimitar syndrome (3; 13%), while two patients (8.7%) had total anomalous pulmonary venous connections. Repair techniques included single patch in 10 patients (43.5%), Warden in 6 (26.1%), and two-patch technique in 4 (17.4%). The median cardiopulmonary bypass and aortic cross-clamp times were 91 and 62 min, respectively. All patients were extubated in the operating room. The median length of hospital stay was 2 days. There were no mortalities or reoperations for pulmonary/systemic venous pathway obstruction. (4) Conclusions: Vertical right axillary thoracotomy is a valuable approach for repairing anomalous pulmonary venous connections with or without sinus venosus defects. All repair techniques, including Warden and scimitar, can be performed safely through this approach. The cosmetic superiority and short hospital stay make this approach worth considering.

## 1. Introduction

Since its introduction in 1957, median sternotomy (MS) has been the gold standard approach for repairing the majority of congenital heart defects (CHDs) in children, offering comprehensive access to the heart and mediastinal structures [1]. However, despite its effectiveness in providing the optimum exposure for conducting cardiopulmonary bypass and performing the necessary repair techniques, MS has several inherent drawbacks, most importantly, postoperative pain, and the long time needed for recovery and return to unrestricted activity. In addition, there are cosmetic disadvantages and the sternotomy scar may have a long-term psychological impact, especially on females [2]. Given this, there is an ongoing need for an alternate approach that is safe, effective, and cosmetically superior to sternotomy.

The vertical right axillary thoracotomy (VRAT) has emerged as a promising minimally invasive alternative. First proposed nearly two decades ago, VRAT is a muscle-sparing mini-thoracotomy incision along the right axilla that provides the necessary exposure to the mediastinal structures needed to perform the repair in a wide variety of CHDs [3]. This approach offers a more cosmetically acceptable outcome by avoiding the highly visible sternotomy scar and has demonstrated comparable clinical results for various CHD repairs [4].

Multiple studies have reported the various benefits of VRAT, which have positioned this approach as a valuable alternative to traditional MS for the repair of many CHDs, even the more complex ones [5]. Despite this data, widespread utilization of this technique has been limited, and it has not been widely adopted, especially in the United States, and for other defects beyond simple atrial septal defects (ASDs). In the current study, we report our VRAT outcomes in repairing anomalous pulmonary venous connections (APVCs) with or without sinus venosus ASDs.

## 2. Materials and Methods

This is a retrospective review of twenty-three consecutive patients that were analyzed during the study period. Approval from the Institutional Review Boards (IRB) at Westchester Medical Center/New York Medical College (IRB# 21396) and at the University of Minnesota (IRB # 00014940) where these patients were treated were obtained.

The study period extended from April 2018 to February 2024. Patients’ demographics and perioperative surgical data as well as latest follow-up information were collected and analyzed.

All pediatric patients (<18 years of age) were included who underwent primary repair of anomalous pulmonary venous connections (APVCs) with or without associated sinus venosus (SV) ASDs through VRAT with a minimum postoperative follow-up time of six months. Those who were excluded were adults (>18 years old) who underwent primary or reoperative MS, had isolated left partial anomalous pulmonary venous connection (PAPVCs) to the left innominate vein, or non-cardiac total anomalous pulmonary venous connections (TAPVCs), or who were lost to follow-up.

Results are given as mean ± standard deviation (SD) and/or median and range when relevant. The median follow-up was 2.9 years (6 months–5.2 years).

## 3. Results

The analysis included 23 patients from two centers who were operated on by the same surgeon (S.M.S). The patients’ characteristics, operative details, and overall outcomes are documented in Table 1. The median age at the time of surgery was 36 months (1–277 months) and the median weight was 14.4 kg (3.6–79.4 kg). More than half of the patients were females (*n* = 13; 56.5%). None of the patients had any chromosomal or genetic abnormalities.

**Figure 1:**
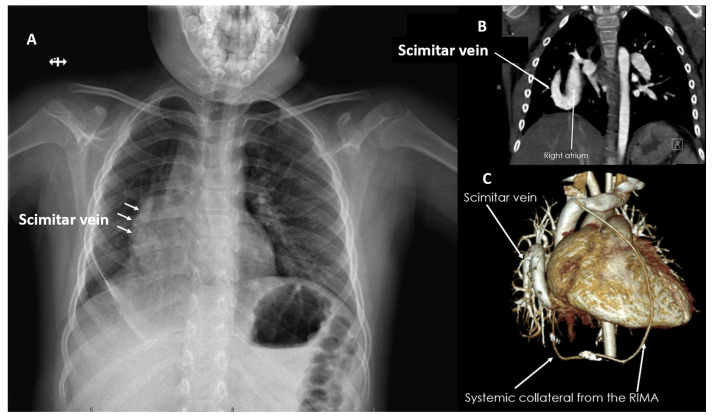
(**A**) A 6-year-old with scimitar syndrome. (**B**) Preoperative chest X-ray showing the scimitar vein, which was confirmed by computed tomography scan (**C**) and the 3D reconstruction showing the abnormal systemic collaterals to the right lung. RIMA: right internal mammary artery.

Diagnostic modalities included routine transthoracic echocardiography (TTE) and when there was doubt about the pulmonary venous connections, cross-sectional imaging confirmation with either computed tomography (CT) or magnetic resonance imaging (MRI) was obtained. The most frequent primary diagnosis was PAPVCs (*n* = 14; 60.9%), while two patients (8.7%) had TAPVC to the coronary sinus (CS). Scimitar syndrome was present in three patients (13%) (Figure 1A–C). SV ASDs with partial anomalous venous return (PAPVR) were present in the remaining four patients (17.4%). Isolated inferior SV defects were found in two patients (8.7%), while the superior type of SV and the CS defects were each present in one patient (4.3%). The indication for surgery was the presence of symptoms and/or right-sided cardiac chamber enlargement.

**Figure 2:**
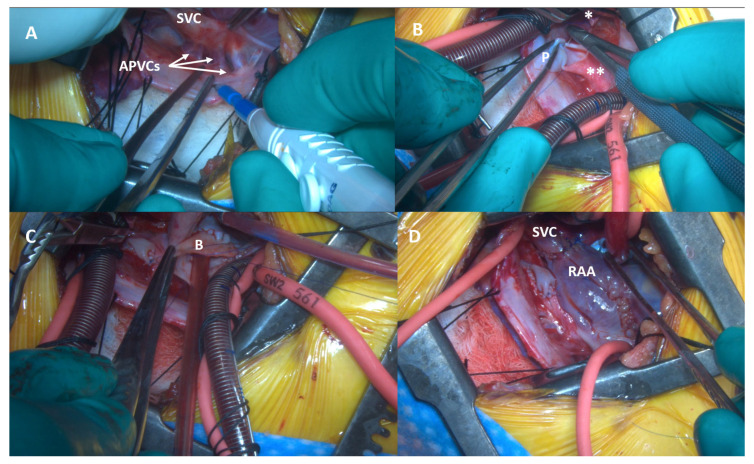
(**A**–**D**): Operative photos demonstrating the Warden procedure through vertical right axillary thoracotomy: (**A**) The view of the anomalous pulmonary venous connections (APVCs) inserting high into the superior vena cava (SVC); (**B**) after initiation of cardiopulmonary bypass and cardioplegic arrest, the superior vena cava is transected above the highest anomalous pulmonary vein and the cardiac end (**) is closed with a bovine pericardial patch (P), while the cranial end (*) will be connected later to the right atrial appendage; (**C**) another pericardial patch is used to create an intra-atrial baffle (**B**), thus directing the anomalous veins to the left atrium through the atrial septal defect. (**D**) The final view is shown with the superior vena cava (SVC) connected to the right atrial appendage (RAA). APVCs: anomalous pulmonary venous connections; SVC: superior vena cava; P: pericardial patch; B: baffle; RAA: right atrial appendage.

**Figure 3:**
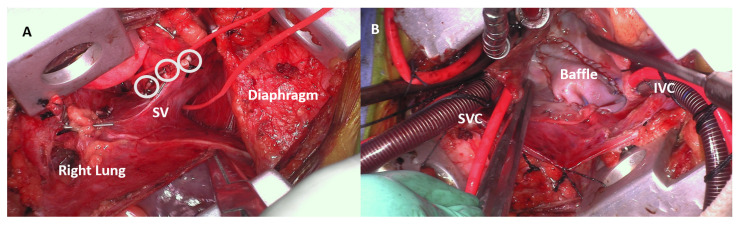
(**A**,**B**): Operative photos of a patient who underwent repair of scimitar syndrome via right axillary thoracotomy, with the scimitar vein (SV) shown in (**A**) encircled with a red vessel loop and the systemic arterial collaterals (white circles) ligated and divided, while in (**B**), the intra-atrial baffle is created to divert the scimitar vein to the left atrium via the atrial septal defect. SV: scimitar vein; SVC: superior vena cava; IVC: inferior vena cava.

All repairs were performed through VRAT with no conversion to sternotomy. Cannulation for cardiopulmonary bypass (CPB) was all central through the same thoracotomy incision, and aortic cross-clamp (AXC) with antegrade cardioplegic arrest was the myocardial protection technique utilized in all patients. Repair techniques included single patch (intra-atrial baffle) in 10 patients (43.5%), and Warden (Figure 2A–D)/modified Warden in 6 (26.1%), while the two-patch technique was used in 4 patients (17.4%). Three patients (13%) underwent repair of scimitar syndrome utilizing a two-patch strategy (Figure 3A,B).

The median AXC and CPB times were 62 (31–196) and 91 min (48–257 min), respectively. All patients were extubated in the operating room. Perioperative pain was managed with an erector spinae catheter inserted by the anesthesia team prior to skin incision in the first 10 patients (43.5%), while later, as we learned from experience, a combination of spinal morphine and erector spinae block was sufficient. Postoperatively, paracetamol and ketorolac were the scheduled pain management routine. The median length of hospital stay was 2 days (1–6 days).

There was no early or late mortality. No patients required permanent pacemaker insertion and there were no phrenic nerve or other neurological injuries. No patients required reoperation for pulmonary or systemic venous pathway obstruction. One patient developed wound seroma nearly a month after the repair and it spontaneously drained with no need for intervention. No other wound-related complications were reported. Two patients (8.7%) who underwent the Warden procedure developed a mild gradient across the translocated superior vena cava (SVC) during their late follow-up, but neither required intervention.

## 4. Discussion

One cannot ignore the drawbacks of median sternotomy. Although it provides the surgeon with sense of safety and a degree of comfort especially when it comes to repairing CHDs in children, it is associated with a longer hospital stay, requires recovery time, rehabilitation, and a longer time to return to unrestricted activity [2,5]. It has also been proven to be cosmetically inferior, and its scar may have long-term psychological effects on both children and their parents.

With the marked improvement in the outcomes of various CHDs in the current era, the search for an alternate approach to sternotomy has become a must. An approach is needed that is cosmetically superior, associated with a shorter length of hospital stay, and, at the same time, offers safety and effectiveness [6]. We demonstrated previously the safety and efficacy of right axillary thoracotomy in repairing a wide variety of CHDs in infants and children [7]. This has become our current approach of choice for non-complex CHDs. Our first experience covered a wide variety of CHDs without targeting a specific lesion. In the current manuscript, we evaluated our outcomes in repairing anomalous pulmonary venous connections with or without sinus venosus ASDs.

In the current study, surgeries were performed at two institutions but by the same surgeon. Our technique has been described previously, but a brief explanation is as follows: All markings are performed with the patient in the supine position before shifting to a modified left lateral decubitus with the right arm abducted above the shoulder. The incision is vertical and ranges between 4 and 5 cm in length in line with the right mid-axillary line. After the creation of generous skin and subcutaneous flaps, the right chest is entered along the third or fourth intercostal space depending on the level of APVCs. For the Warden procedure, entry is made through the third intercostal space and either a left innominate vein or a high SVC cannulation is performed. In all cases, myocardial protection was provided with antegrade cardioplegia after application of the AXC. The repair technique is the same that performed in sternotomy. The current study included 23 patients with a median age of 36 months (range 1–277 months) and median weight of 14 kg (range: 3.6–79.4 kg). The most common abnormality was PAPVCs (14 patients; 60.9%). We had two patients (8.7%) who had TAPVCs to the coronary sinus, while scimitar syndrome was present in three patients (13%). A total of seven patients (30.4%) had SV defects in the current study. In a study by Rao and colleagues, the authors performed VRATs in 14 patients for repairing SV defects and PAPVCs. Their median age and weight were 9.5 years and 21 kg, respectively [8]. However, the youngest patient in their series was a 5-year-old and the lowest weight was 14 kg. They used peripheral venous cannulation for CPB via percutaneous cannulation of the right internal jugular and femoral veins. We used central cannulation in all of our patients, a strategy that we insisted upon to minimize risks associated with peripheral cannulation. They used the two-patch repair technique in all of their patients. We have demonstrated, in the current study, the ability to perform all PAPVC repair techniques via VRAT. In our cohort, single-patch, two-patch, and Warden procedures were performed in 10 (43.5%), 4 (17.4%), and 6 patients (26.1%), respectively. Three patients in our series underwent repair of scimitar syndrome using the two-patch technique as well.

Our median AXC and CPB times were 62 and 91 min, respectively. The AXC and CPB times in Rao and colleagues’ series were 52 and 70 min, respectively, but this was for the two-patch repair technique only.

We had no conversion to sternotomy and no early/late mortalities or reoperations. No patient developed pulmonary or systemic venous pathway obstruction but two (8.6%) had a mild gradient across the translocated SVC when the Warden procedure was used.

A recent comparison between right mini-thoracotomy and median sternotomy for repairing PAPVCs and SV defects included 83 patients [9]. This study included both children and adults and the authors performed only the double-patch technique for repair. The authors avoided peripheral cannulation in small children, and they did not cannulate the SVC directly, but used a venous cannula through the right atrial appendage that was directed high into the SVC. They also used fibrillatory arrest in the majority of pediatric patients. The authors found significantly lower chest tube drainage in the minimally invasive group in children. One patient in the minimally invasive group in children had mild SVC stenosis that did not require reintervention. The length of hospital stay was significantly shorter in the minimally invasive group (4.40 ± 0.70 vs. 4.94 ± 0.97; *p* = 0.04). They concluded that the minimally invasive approach can be a safe and practical alternative to sternotomy with better cosmetic and psychological outcomes. The authors of this study performed minithoracotomy far enough from the nipple so as to not disturb future development of the breast tissue, but we believe this is something that is hard to predict with certainty, especially in prepubescent girls, and we believe that VRAT is a better approach for this particular reason, as it is performed far enough from the breast tissue and hidden under the right arm in almost all patients.

**Figure 4:**
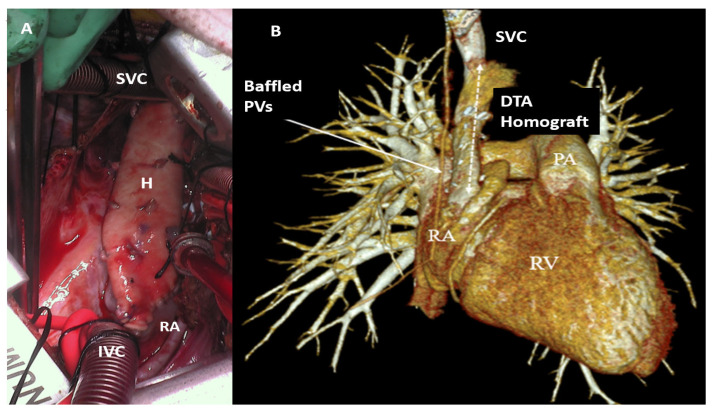
(**A**,**B**): (**A**) Operative photo of right axillary thoracotomy showing our modified Warden technique with the use of an aortic homograft (H) for translocating the superior vena cava (SVC) to the right atrium (RA); (**B**) follow-up scan showing the unobstructed superior vena cava pathway and the baffled pulmonary veins to the left atrium. SVC: superior vena cava; IVC: inferior vena cava; H: homograft; RA: right atrium; PVs: pulmonary veins; DTA: descending thoracic aortic; RV: right ventricle; PA: pulmonary artery.

We have previously demonstrated the Warden procedure via VRAT. In older children and teenagers, we have moved away from direct translocation of the SVC to the right atrial appendage to avoid development of late anastomotic stenosis, which has been the Achilles’ heel of this operation. We use a segment of aortic homograft as an interposition graft to connect the SVC to the right atriotomy in a manner similar to the technique used for a right ventricular-to-pulmonary artery conduit [10]. This creates a large unobstructed pathway from the SVC to the right atrium (Figure 4A,B).

Despite the demonstrated clear advantages of the right axillary thoracotomy, the adoption of this approach especially in the United States, has been very slow, with ongoing concerns related to safety, quality of repairs, and outcomes. A recent landmark multi-institutional study that included over 3000 patients from 11 centers demonstrated that minithoracotomy incisions provide safe access that allows excellent-quality repair of a large variety of CHDs [11]. This study included infants, children, and adults, with the lowest patient weight being 3.1 kg. In this study, the most common defect was ASD (2211 patients; 73.5%), and 466 patients (15.5%) had SV defects with PAPVCs. Scimitar syndrome was present in two patients (0.1%) and TAPVCs in three patients (0.1%). Taking into consideration the multi-institutional nature in this large study, and the various approaches and techniques used, the overall outcomes were excellent, with no surgical mortality. In terms of SV/PAPVCs defects, one patient underwent SVC stent placement 3 years after a double-patch repair of PAPVCs.

Indeed, right axillary thoracotomy has been proven to be safe and effective and we believe it is here to stay [12].

## 5. Conclusions

In conclusion, VRAT is a valuable approach to consider for repairing various types of anomalous pulmonary venous connections. It is associated with satisfactory outcomes and superior cosmetic results, and the quality of repair is excellent; it has the ability to address various types of abnormal pulmonary venous connections including scimitar syndrome.

## 6. Study Limitations

The current study is limited by its retrospective nature and the small number of patients included. We did not include a control group, but the inclusion and exclusion criteria were clarified to determine the suitability of the patients for the VRAT approach. Longer-term follow-up with a larger group of patients is needed to continue documenting the value of VRAT in addressing this particular heart defect.

## Figures and Tables

**Table 1:** Characteristics and outcomes of the current cohort.

Age: Median (range)	36 months (1–277 months)
Weight: Median (range)	14.4 kg (3.6–79.4 kg)
Female: (%)	13 (56.5%)
Primary Diagnosis	
Partial APVCs (%)	14 (60.9%)
Scimitar Syndrome (%)	3 (13%)
Total APVCs to Coronary Sinus (%)	2 (8.7%)
Conversion from VRAT to Sternotomy	0
Repair Technique	
Single Patch (%)	10 (43.5%)
Warden (%)	6 (26.1%)
Two-Patch (%)	4 (17.4%)
Scimitar Repair (%)	3 (13%)
Intraoperative	
CPB Median (range)	91 min (48–257 min)
AXC Median (range)	62 min (31–196 min)
Mortality	
Early/Late Mortality	0
Late Follow-Up	
Pulmonary/Systemic Venous Pathway Obstruction	0
Warden Mild SVC Gradient (%)	2 (8.7%)

## Data Availability

All data are available within the manuscript.

## Abbreviations

The following abbreviations are used in this manuscript:

APVCs	anomalous pulmonary venous connections
ASD(s)	atrial septal defect(s)
AXC	aortic cross-clamp
CPB	cardiopulmonary bypass
CS	coronary sinus
CT	computed tomography
IRB	institutional review board
MRI	magnetic resonance imaging
PAPVCs	partial anomalous pulmonary venous connections
PAPVR	partial anomalous pulmonary venous return
SD	standard deviation
SV	sinus venosus
SVC	superior vena cava
TAPVCs	total anomalous pulmonary venous connections
TEE	transesophageal echocardiography
TTE	transthoracic echocardiography
VRAT	vertical right axillary thoracotomy
